# Supplementary material for: The longitudinal relation between adolescents’ learning outcomes and internalizing symptoms: The role of ADHD symptoms
Source: PLoS One. 2026 May 4;21(5):e0347582. doi: 10.1371/journal.pone.0347582 (PMC13138621; doi:10.1371/journal.pone.0347582)
Supplement: S1 Table — (DOCX) [file pone.0347582.s001.docx]

**S1 Table**

*Sample Sizes Separately per Time Point, Variable, and by Gender*

| Time point | Gender | School records | Depression,  Anxiety,  ADHD symptoms |
| --- | --- | --- | --- |
| 0 | Female | 5,270 | 5,672 |
|  | Male | 5,685 | 6,188 |
|  | Total | 10,955 | 11,860 |
| 1 | Female | 5,016 | 5,341 |
|  | Male | 5,436 | 5,861 |
|  | Total | 10,452 | 11,202 |
| 2 | Female | 4,877 | 3,850 |
|  | Male | 5,389 | 4,231 |
|  | Total | 10,266 | 8,081 |
| 3 | Female | 4,476 | 3,645 |
|  | Male | 4,927 | 4,013 |
|  | Total | 9,403 | 7,658 |
