# Supplementary material for: The longitudinal relation between adolescents’ learning outcomes and internalizing symptoms: The role of ADHD symptoms
Source: PLoS One. 2026 May 4;21(5):e0347582. doi: 10.1371/journal.pone.0347582 (PMC13138621; doi:10.1371/journal.pone.0347582)
Supplement: Attachment — Submitted filename: Peer Review of Manuscript PONE_PlosOne.docx [file pone.0347582.s002.docx]

**Peer Review of Manuscript PONE-D-25-47753**

Dear Authors,

Thank you for the opportunity to review your manuscript, "The longitudinal relation between adolescents’ learning outcomes and internalizing symptoms: The role of ADHD symptoms". This is an important and timely article that addresses a complex and highly relevant issue. The use of the impressive ABCD Study dataset and the application of sophisticated latent change score models are significant strengths.

This note is to explain the reasoning behind my answers to the review questions and to provide detailed feedback for revision. While the manuscript is clearly written and the statistical analyses are appropriate and rigorous, I have indicated that the data only partly support the conclusions at this stage. This assessment is based on several key areas that, I believe, require revision to strengthen the manuscript's overall impact and clarity. My detailed comments are provided below.

**Major Points:**

1. **Operationalization and Measurement of "Learning Outcomes":** The sole measure for learning outcomes is a single parent-report item on their child's school grades. While acknowledging the constraints of large-scale datasets, this is a significant limitation that impacts the strength of the conclusions drawn about this core construct.
   - **Recommendation:** Please expand the discussion on this limitation. Specifically, address the potential biases of parent-reported grades versus more objective measures and discuss the implications of treating this ordinal variable as continuous in the models. Furthermore, for consistency, consider changing the keyword "academic achievement" to "parent-reported school grades" or "school records," which are the terms used more consistently throughout the manuscript.
2. **Interpretation of Key Findings:** The manuscript presents several significant and compelling gender differences in the results, but these are not interpreted or contextualized in the discussion. This omission leaves a major part of the findings unexplored.
   - **Recommendation:** The Discussion section should be expanded to include an interpretation of these gender differences. What are the potential theoretical or clinical implications of these findings? For instance, the finding that females show a larger decrease in both anxiety and depression symptoms warrants further exploration in the context of existing literature on gender differences in adolescent mental health.
3. **Contextual Factors:** The potential confounding impact of the COVID-19 pandemic on the developmental trajectories, particularly for depression, is noted as a limitation but is not sufficiently integrated into the main interpretation of the results.
   - **Recommendation:** The authors should integrate this potential confounder more deeply into the interpretation of the results in the Discussion. This could help explain why the depression trajectory results contradicted both the authors' hypotheses and previous literature.
4. **Clarity of Hypothesis Testing Regarding ADHD's Role:** The third research question hypothesized that ADHD symptoms would explain the relation between school records and internalizing symptoms. As the bivariate analyses found no longitudinal coupling between them, this hypothesis could not be directly tested. The current framing might be slightly misleading.
   - **Recommendation:** Consider reframing the third research question and hypotheses in the Introduction. Instead of framing ADHD as an explanatory variable for a presumed relationship, it would be more accurate to state that the study aims to simultaneously examine the trivariate dynamic relationships among the three constructs.

**Minor Points:**

1. **Framing in the Introduction:** The introduction begins by discussing Specific Learning Disorder (SLD), but the study focuses on a continuum of symptoms in a general population sample.
   - **Recommendation:** Please make the transition from the discussion of SLD to the study's focus on the continuum of learning outcomes smoother and more explicit.
2. **Data from T4:** The methods section states that data from T4 were excluded because academic achievement data were missing.
   - **Recommendation:** Please clarify if data for the other key variables (e.g., CBCL) were available at T4 and briefly justify the decision to stop at T3 for all variables rather than using models that can accommodate missing data.
3. **Reporting of Model Fit:** In several instances, model selection relied on the chi-squared difference test, even when an alternative model had similar or better fit indices (e.g., RMSEA/CFI).
   - **Recommendation:** It would be helpful to add a brief sentence justifying the reliance on the chi-squared difference test in these specific cases, especially given the large sample size.

I believe that by addressing these comments, the manuscript will be an excellent contribution to the journal.
